# Commentary on: Complications of Capsulectomies: An Analysis of the American College of Surgeons National Surgical Quality Improvement Program Database

**DOI:** 10.1093/asjof/ojac026

**Published:** 2022-04-11

**Authors:** Patricia McGuire

See the Original Article here.

The number of capsulectomies performed has increased since 2019.^1^ This paper, “Complications of Capsulectomy: An Analysis of the American College of Surgeons National Surgical Quality Improvement Program (NSQIP) Database,” reviews the complications reported by participating institutions for surgeon-reported capsulectomy procedures.^2^ The authors state that this is the first estimate of the incidence of complications from capsulectomy, and from my review of the literature, they seem to be correct. They report a minor complication rate of 0.8% and a major complication rate of 2.4%. The limitations of the paper include the lack of specificity of the procedure performed, whether total or partial capsulectomy, and whether the implants were subglandular or submuscular, which could affect the rate of complications. Approximately, 16.7% of patients had a concomitant major procedure, and it is unknown to which procedure the complication was related. A paper published in *Aesthetic Surgery Journal* which reviewed CosmetAssure (Birmingham, AL) data for complications of capsulectomy reported a similar major complication rate of 2.8%, with twice the complication rate of revisional procedures performed without a capsulectomy.^3^ This paper shows that capsulectomy is not without risk, and further studies are indicated to determine the true incidence so that patients considering capsulectomy can make an informed decision.
